# Lack of association between *rrl* and *erm*(41) mutations and clarithromycin resistance in *Mycobacterium abscessus* complex

**DOI:** 10.1590/0074-02760170080

**Published:** 2017-11

**Authors:** Maiara dos Santos Carneiro, Luciana de Souza Nunes, Simone Maria Martini de David, Afonso Luis Barth

**Affiliations:** 1 Universidade Federal do Rio Grande do Sul, Faculdade de Farmácia, Programa de Pós-Graduação em Ciências Farmacêuticas, Porto Alegre, RS, Brasil; 2 Universidade Federal do Pampa, Uruguaiana, RS, Brasil; 3 Centro de Pesquisa Experimental, Hospital de Clínicas de Porto Alegre, Laboratório de Pesquisa em Resistência Bacteriana, Porto Alegre, RS, Brasil; 4 Laboratório Central de Saúde Pública, Porto Alegre, RS, Brasil

**Keywords:** clarithromycin resistance, *erm*(41) gene, *rrl* gene, *M. abscessus* complex, inducible resistance, acquired resistance

## Abstract

**BACKGROUND:**

*Mycobacterium abscessus* complex (MABC) includes species with high resistance rates among mycobacterial pathogens. In fact, MABC infections may not respond to clarithromycin treatment, which has historically been very effective against MABC infection. Molecular markers have been proposed to detect both acquired (*rrl* polymorphisms) and inducible (*erm*(41) polymorphisms) clarithromycin resistance in MABC isolates.

**OBJECTIVES:**

This study aimed to evaluate the susceptibility profile and molecular markers of clarithromycin resistance in MABC.

**METHODS:**

The clarithromycin susceptibility profile was determined by broth microdilution with reads on days 3, 5, 7 and 14. Mutations in the *rrl* and *erm*(41) genes were evaluated by polymerase chain reaction (PCR) using specific primers, followed by sequencing.

**FINDINGS:**

A total of 14 *M. abscessus* subsp. *abscessus* isolates and 28 *M. abscessus* subsp. *massiliense* isolates were evaluated, and clarithromycin resistance was observed in all isolates for up to three days of incubation. None of the 42 isolates exhibited a point mutation in the *rrl* gene, while all the isolates had a T28 polymorphism in the *erm*(41) gene. Moreover, all 28 *M. abscessus* subsp. *massiliense* isolates had a deletion in the *erm*(41) gene.

**MAIN CONCLUSIONS:**

While all the MABC isolates exhibited acquired clarithromycin resistance, no isolates exhibited a point mutation in the *rrl* gene in this study. The *M. abscessus* subsp. *massiliense* isolates demonstrated clarithromycin resistance, which is an uncommon phenotype. The molecular data for the *rrl* and *erm*(41) genes were not consistent with the phenotypic test results of clarithromycin susceptibility, indicating a lack of correlation between molecular clarithromycin resistance markers for both acquired and inducible resistance.

Several rapidly growing mycobacteria (RGM) can cause infections in humans, most of which are caused by *Mycobacterium abscessus* complex (MABC), *M. chelonae* and *M. fortuitum* (Brown-Elliott et al. 2002a, b). MABC is comprised of three closely related subspecies (*M. abscessus* subsp. *abscessus, M. abscessus* subsp. *bolletii* and *M. abscessus* subsp. *massiliense*), which are the most pathogenic and multidrug-resistant (MDR) isolates among all RGMs (Griffith et al. 2015, Lee et al. 2015). Infections due to MABC are difficult to treat (Kasperbauer & de Groote 2015, Kang & Koh 2016) because they are intrinsically resistant to not only classical antituberculosis drugs but also to most antibiotics that are currently available (Ryu et al. 2016, Stout et al. 2016).

In fact, clarithromycin has become the drug of choice for infections caused by MABC, and it was the treatment mainstay until induced resistance was described (Griffith et al. 2007, Nash et al. 2009). Inducible resistance is believed to be mainly related to the capacity of some MABC subspecies to encode a functional erythromycin ribosomal methylase gene, *erm*(41), which modifies the binding site for macrolide antibiotics, resulting in inducible macrolide resistance (Bastian et al. 2011, Maurer et al. 2014). Because resistance to clarithromycin is increasing, its use as a monotherapy for treatment is not recommended, and it should instead be combined with other drugs (Kasperbauer & de Groote 2015). Additionally, sequence analysis of the *erm*(41) gene allowed for the identification of *M. abscessus* subspecies (Griffith et al. 2015).

Acquired resistance to clarithromycin is related to point mutations in a region of the *rrl* gene encoding the peptidyltransferase domain of 23S rRNA (Nash et al. 2009). The main molecular mechanism of clarithromycin-acquired resistance reportedly occurs through adenine point mutations at either position 2058 (A2058G) or position A2059 in the 23S rRNA gene (Maurer et al. 2012).

Phenotypic tests (susceptibility profile) revealed that acquired resistance can be detected for up to five days of incubation of the MABC with clarithromycin, while inducible resistance requires prolonged incubation (14 days of incubation) (Bastian et al. 2011).

The aim of this study was to evaluate correlation between the susceptibility profile and the molecular mechanisms of clarithromycin resistance in MABC.

## MATERIALS AND METHODS

A total of 42 isolates from a previous surveillance study (Nunes et al. 2014) of samples from different sources collected between 2007 and 2013 were used in this study. The susceptibility profile was determined by broth microdilution following the guidelines of the Clinical and Laboratory Standards Institute [CLSI document M24-A2 (CLSI 2011)] with readings on days three, five, seven and 14. Point mutations in the *rrl* gene were evaluated by polymerase chain reaction (PCR) amplification with primers 18 (AGT CGG GAC CTA AGG CGA G) and 21 (TTC CCG CTT AGA TGC TTT CAG) (Meier et al. 1994). Polymorphisms in the *erm*(41) gene were evaluated with primers ERM1f (CGC CAA CGA GCA GCT CG) (Bastian et al. 2011) and MC823 (GAC TTC CCC GCA CCG ATT CCA C) (Nash et al. 2009) using the following steps: 94°C for 5 min, 40 cycles of 94°C for 1 min, 64°C for 1 min and 72°C for 1 min, and 72°C for 10 min. *M. abscessus* ATCC 19977 was used as quality control.

Genetic profiling of the two genes was performed by partial sequencing using the ABI 3500 Genetic Analyser with 50-cm capillaries and the POP7 polymer (Applied Biosystems, Thermo Fisher Scientific, Califórnia, EUA). PCR products were labelled with 3.2 pmol of specific primers and 1 μL of the BigDye Terminator v3.1 Cycle Sequencing Kit (Applied Biosystems) in a final volume of 10 μL. Sequence alignment was performed using the program Biological Sequence Alignment Editor - BioEdit 7.2-5. Homology analysis was performed by comparing the consensus sequences obtained for each isolate with those deposited in GenBank using the BLAST algorithm (Basic Local Alignment Search Tool, http://www.ncbi.nlm.nih.gov/BLAST). The erm(41) sequences of *M. abscessus* subsp. *Abscessus* T28 sequevar ATCC19977 (GenBank accession number HQ127365) and *M. abscessus* subsp. *Massiliense* CIP108297 (GenBank HQ127368) were used as references. This study was approved by the Ethics Committee of “Grupo de Pesquisa e Pós-Graduação (GPPG)” of “Hospital de Clínicas de Porto Alegre” (reference number CAAE: 35684114.5.0000.5327).

## RESULTS

A total of 14 isolates belonging to *M. abscessus* subsp. *Abscessus* and 28 isolates belonging to *M. abscessus* subsp. *Massiliense* were identified by analysing the *erm*(41) gene.

Clarithromycin resistance was observed in all the isolates for up to three days of incubation.

For all the isolates, PCR yielded amplicons of the expected size (1500 bp) for the *rrl* gene; however, none of the 42 isolates exhibited point mutations at positions 2058 or 2059 in the peptidyltransferase region of 23S rRNA (*rrl* gene), which is the gene related to acquired clarithromycin resistance. For the *erm*(41) gene, the PCR reactions generated fragments of 764 bp for *M. abscessus* subsp. *Abscessus* and smaller amplicons (~250 bp) for all the *M. abscessus* subsp. *Massiliense* isolates. These isolates also presented the T/C polymorphism at the 28th nucleotide of *erm*(41), corresponding to a tryptophan at the 10th codon. Moreover, all *M. abscessus* subsp. *massiliense* isolates had a deletion in *erm*(41) compared to the reference sequence.

Interestingly, point mutations were identified at other positions in the *erm*(41), gene but they did not correspond to amino acid alteration (silent mutations) (Table).

**TABLE:** Positions of nucleotide substitutions in *erm(*41) gene

	Isolate	Position	Point mutation	Specie
*erm*(41)	131253 121187	120	A - G	*M. abscessus* subsp. *abscessus* *a*
	110920 900769	159	T - C	
	131253 130295	238 255	A - G C - A	*M. abscessus* subsp. *abscessus* *a*
	121187 900161	330	A - C	
	130295 121187 900161	275 336	G - T T - C	*M. abscessus* subsp. *abscessus* *a*
		67	C - T	
	800107	184 204	C - A A - G	*M. abscessus* subsp. *massiliense* *b*
		218	C - T	

a the gene sequence of *Mycobacterium abscessus* subsp. *abscessus* ATCC 19977 (GenBank accession number HQ127365) was used as reference;

b the gene sequence of *M. abscessus* subsp. *massiliense* strain CIP108297 (GenBank HQ127368) was used as reference.

## DISCUSSION

Widespread use of macrolides in clinical medical care has created favourable conditions for the selection of resistant RGM isolates (Bailey et al. 2008). The two main mechanisms of macrolide resistance are drug-efflux pumps and modification of ribosomal drug target sites via *erm* methylase expression (Nessar et al. 2012). This modified *erm* prevents binding of the antibiotic to the ribosome by mono- or di-methylation, which disturbs the shape and chemical makeup of the drug binding site, thereby reducing the affinity of the macrolide to the ribosome (Douthwaite et al. 2005, Mougari et al. 2016b). *Erm* expression is highly regulated, as it is induced in the presence of macrolides; however, its expression is not favourable for the cell because it is associated with significant fitness costs (Brierley 2013).

Nash et al. (2009) and Kim et al. (2010) investigated several isolates of the *M. abscessus* complex and found that *M. abscessus* subsp. *massiliense* was susceptible to clarithromycin, while the susceptibility profile of *M. abscessus* subsp. *abscessus* was variable. Conversely, we found a high percentage of *M. abscessus* subsp. *massiliense* isolates that exhibited clarithromycin resistance, which was also reported by Mougari et al. (2016b). This susceptibility profile difference may be related to local or regional characteristics of MABC isolates. It should be noted that most of the isolates used in our study belonged to the same clone (data not shown), although they were originated from several outbreaks in different cities in southern Brazil (Nunes et al. 2014).

Molecular profiles of the MABC isolates established by Nash et al. (2009) and Kim et al. (2010) revealed the T28 polymorphism of *erm*(41) to be related to resistance and the C28 polymorphism to be related to susceptibility. Therefore, the T28 polymorphism in *M. abscessus* subsp. *abscessus* could be considered a clarithromycin resistance marker. In our study, all 14 isolates of *M. abscessus* subsp. *abscessus* exhibited a T28 polymorphism. It should be noted that in the study by Kim et al. (2010), no prolonged incubation periods were evaluated (microdilution reads were made on the 5th day of incubation).

Regarding *M. abscessus* subsp. *massiliense*, the T28 polymorphism is not necessarily related to clarithromycin inducible resistance, as position 28 is located upstream of the deletion that leads to resistance. However, several *M. abscessus* subsp. *massiliense* isolates with a functional *erm*(41) gene have been reported (Gray et al. 2014). Moreover, two *M. abscessus* subsp. *massiliense* isolates with a functional *erm*(41) gene showing inducible clarithromycin resistance after 14 days have been described (Shallom et al. 2013).

Regarding the molecular basis of acquired resistance, sequencing of the *rrl* gene showed an absence of mutations (wild type) at positions 2058 and 2059 of 23S rRNA in all the isolates analysed in this study. However, we found isolates that exhibited resistance for up to three days of incubation, indicating that another molecular mechanism may be involved in acquired resistance. Although *rrl* wild-type isolates resistant to clarithromycin are not an usual finding, Maurer et al. (2012) described the presence of resistant isolates with no mutations in the *rrl* gene in the MABC. Therefore, mutations solely in 23S rRNA cannot be used as a marker for acquired resistance to clarithromycin.

Because Esteban et al. (2009) demonstrated low minimum inhibitory concentration (MIC) for *erm*(41)-positive isolates and high MIC for *erm*(41)-negative isolates, presence of the *erm*(41) gene is not necessarily correlated with clarithromycin resistance (higher MIC).

In this study, point mutations in the *erm*(41) gene were evidenced, but this exchange of nucleotides did not lead to amino acid alteration. According to Brown-Elliott et al. (2015), only the T28C substitution resulted in loss of *erm*(41) gene function, and no other nucleotide substitution is known to be associated with macrolide susceptibility. This finding disagrees with that of the Kim et al. (2016) study, which demonstrated that *M. abscessus* subsp. *abscessus* has a C-to-T mutation at position 19 (C19 → T), which leads to an Arg → stop codon mutation at codon seven of the *erm*(41) gene and results in loss of *erm*(41) gene function.

The main finding of our study was the high number of *M. abscessus* complex isolates with resistance to clarithromycin that did not correlate with mutations in the *rrl* gene. Therefore, the results of *erm*(41) and *rrl* sequencing should not be considered fully concordant with phenotypic clarithromycin susceptibility tests.
